# Emerging trends and hot spots of sleep and genetic research: a bibliometric analysis of publications from 2002 to 2022 in the field

**DOI:** 10.3389/fneur.2023.1264177

**Published:** 2023-11-08

**Authors:** Ying Tao, Yi Qin, Sifan Chen, Tian Xu, Junhui Lin, Diansan Su, Weifeng Yu, Xuemei Chen

**Affiliations:** ^1^Department of Anesthesiology, Renji Hospital, Shanghai Jiao Tong University School of Medicine, Shanghai, China; ^2^Key Laboratory of Anesthesiology (Shanghai Jiao Tong University), Ministry of Education, Shanghai, China

**Keywords:** sleep, gene, GWAS, CiteSpace, VOSviewer, bibliometric analysis, co-citation analysis

## Abstract

**Background:**

Sleep is an important biological process and has been linked to many diseases; however, very little is known about which and how genes control and regulate sleep. Although technology has seen significant development, this issue has still not been adequately resolved. Therefore, we conducted a bibliometric analysis to assess the progress in research on sleep quality and associated genes over the past 2 decades. Through our statistical data and discussions, we aimed to provide researchers with better research directions and ideas, thus promoting the advancement of this field.

**Methods:**

On December 29, 2022, we utilized bibliometric techniques, such as co-cited and cluster analysis and keyword co-occurrence, using tools such as CiteSpace, VOSviewer, and the Online Analysis Platform of Literature Metrology (http://bibliometric.com/), to conduct a thorough examination of the relevant publications extracted from the Web of Science Core Collection (WoSCC). Our analysis aimed to identify the emerging trends and hot spots in this field while also predicting their potential development in future.

**Results:**

Cluster analysis of the co-cited literature revealed the most popular terms relating to sleep quality and associated genes in the manner of cluster labels; these included genome-wide association studies (GWAS), circadian rhythms, obstructive sleep apnea (OSA), DNA methylation, and depression. Keyword burst detection suggested that obstructive sleep apnea, circadian clock, circadian genes, and polygenic risk score were newly emergent research hot spots.

**Conclusion:**

Based on this bibliometric analysis of the publications in the last 20 years, a comprehensive analysis of the literature clarified the contributions, changes in research hot spots, and evolution of research techniques regarding sleep quality and associated genes. This research can provide medical staff and researchers with revelations into future directions of the study on the pathological mechanisms of sleep-related diseases.

## 1. Introduction

Sleep is a fundamental biological process that is conserved across a wide range of organisms from invertebrates to vertebrates, but the pathobiology and molecular mechanisms of sleep are still not fully understood. There was a strong interest in the genetics of sleep. In the 1930s, Geyer conducted a twin study in children and first showed that genetic factors were involved in the regulation of sleep. Since then, numerous high-quality studies on gene expression profiling indicate that there is a shared genetic basis for sleep across different species (1, 2). In other words, there is a belief that the mechanism responsible for sleep is evolutionarily conserved and that similar genetic pathways are involved in regulating sleep across different organisms. Nowadays, various new programs and techniques have been developed, leading to breakthroughs in the understanding of sleep function, circadian rhythms, and pathological mechanisms of sleep-related diseases (3–5).

However, the molecular and pathobiology mechanisms of sleep and sleep-related diseases are quite complex and least understood phenomena for a long time. Genetic analysis of sleep has only become a significant discipline in the past decade, with the focus expanding to drosophila, zebrafish, and worms. With the help of genetics, many sleep-related problems have been solved, for example, the identification of loci related to various sleep disorders such as obstructive sleep apnea (OSA), major depressive disorder (MDD), and insomnia disorder. As genetic technologies continue to develop, genes will be a powerful tool in solving these problems.

In this study, we utilized bibliometric citation analysis, which is widely employed to map and analyze the performance of specific fields, to gain insights into the temporal trends in the field of sleep quality and associated genes. It provides valuable predictions about the progress and development of specific fields based on factors such as publication volume and citation frequency (6, 7). The published studies have played a significant role in advancing specific research fields (8). To recognize the significance of the sleep and genetics topic and the effectiveness of bibliometric analysis, we conducted an analysis of publications related to this theme. Our objective was to assist researchers to identify the current trends and hot spots in this field, both in the past and future and to provide a comprehensive understanding of the evolution of this field.

## 2. Methods

### 2.1. Data source and search strategy

**Figure 1:**
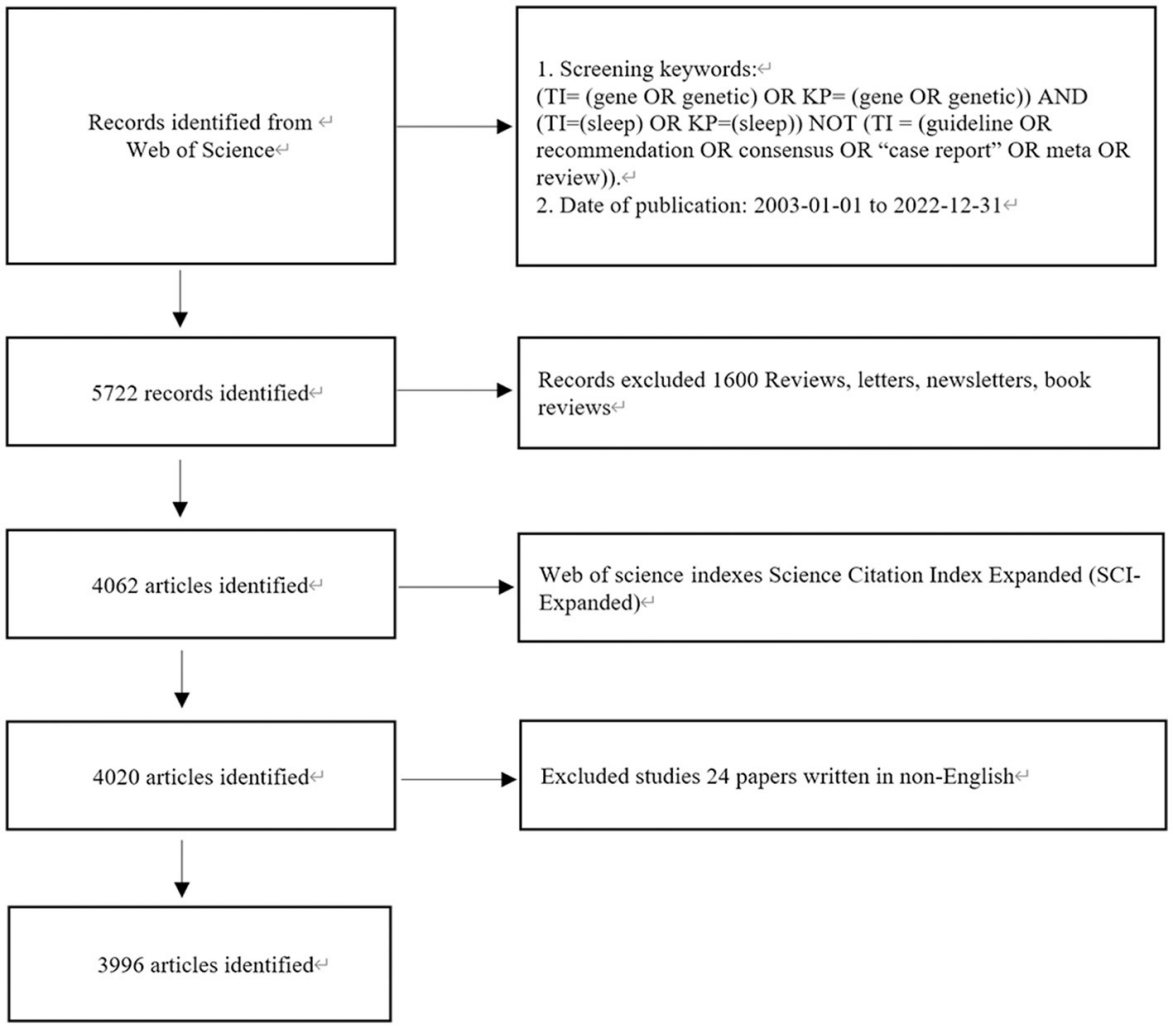
Flow diagram of the inclusion process. The detailed process of screening and enrollment.

The Web of Science core collection database was searched by retrieval form (TI = (gene OR genetic) OR KP = (gene OR genetic)) AND (TI = (sleep) OR KP=(sleep)) NOT (TI = (guideline OR recommendation OR consensus OR “case report” OR meta OR review)). The retrieval time was limited to January 01, 2003 to December 31, 2022. The inclusion criteria were studies related to the search, excluding reviews, letters, briefings, book reviews, etc., resulting in 3,996 articles. The data were used for visual analysis of authors, institutions, countries, journals, co-cited literature, and keywords. The detailed process of enrollment and screening is shown in Figure 1.

### 2.2. Data selection

We used Web of Science (https://wcs.webofknowledge.com) to analyze search results and create a histogram that illustrates publication trends. We then converted the WoSCC data to the UTF-8 format and utilized the “total literature analysis” option to analyze publication trends across different countries and the “partnership analysis” option to analyze intercountry/regional relationships. Additionally, we utilized Web of Science to extract the histogram that displays the publication trend and analyze the research status, hot spots, and trend with the above software.

### 2.3. Data analysis and visualization

The publications' complete records and cited references were obtained from the WoSCC database and saved as a text file. We used VOSviewer1.6.18 (version 1.6.18, Leiden University, van Eck NJ and Waltman L), which can provide three kinds of visualization maps: network maps, overlay maps, and density maps, to analyze the information of authors, institutions, journals, countries, references, and keywords. We used CiteSpace 6.1.6 to calculate keywords burst and reference co-citation analysis. The graphs were created by the abovementioned software, and the layout was adjusted by Pajek and SCImago Graphica by using the above software to draw a visual map and analyze the research status, hot spots, and trend of sleep quality and associated genes.

## 3. Results

### 3.1. Analysis of publications and time trends

**Figure 2:**
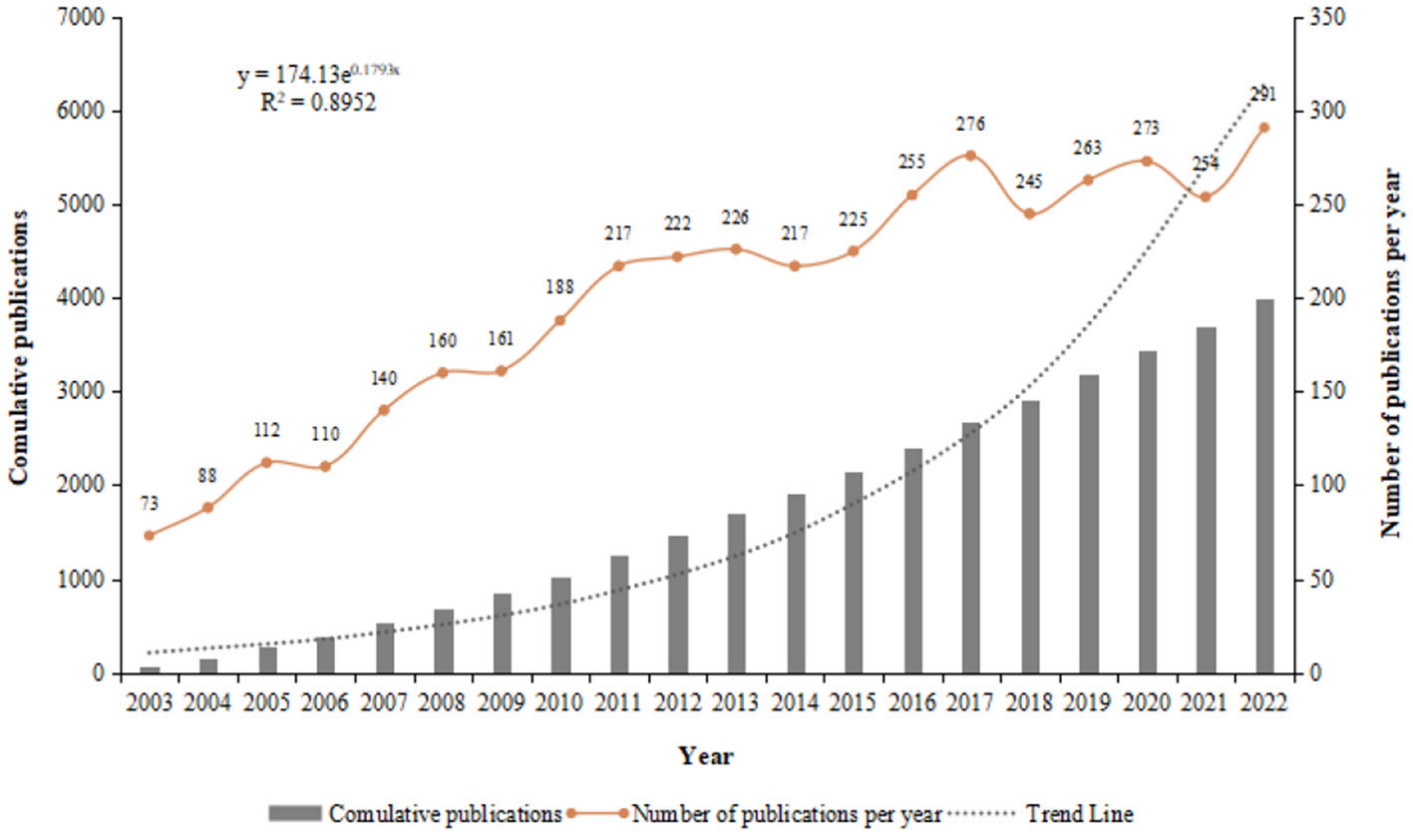
Annual and total number of publications on sleep.

Over the past 20 years, researchers have contributed 4,000 articles to the field of sleep, including editorial materials and reviews. The number of sleep-related publications has shown consistent growth, increasing from 73 in 2003 to 291 in 2022, while three minor dips in publication rates occurred from 2013 to 2014, from 2017 to 2018, and from 2020 to 2021. In 2022, the number of articles on sleep reached a peak at 291, with a significant increase of 37 publications from the previous year (Figure 2).

### 3.2. Analysis of countries and institutions

**Figure 3:**
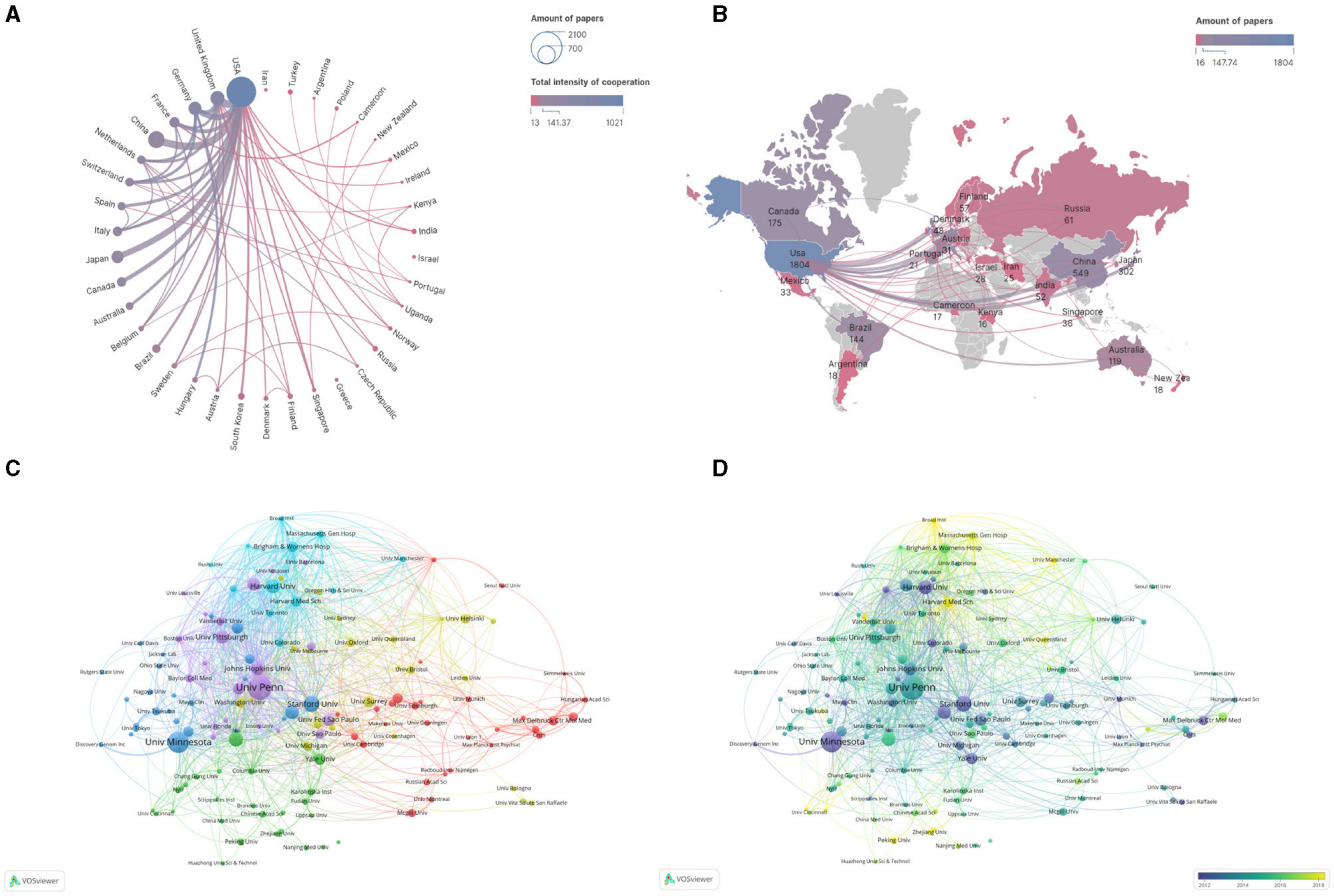
Cooperation maps among countries and institutions. **(A)** Cooperation relationships of countries. The size of points means the number of publications. The colors and the weight of the lines mean the intensity of cooperation. **(B)** World map of cooperation relationships among countries. The colors show the number of publications. **(C)** Cooperation relationships among institutions. Colors represent clusters automatically calculated by VOSviewer. **(D)** Time trend of cooperation among institutions. The colors correspond to the annual time periods of publications.

We analyzed the countries and institutions of the authors of these articles. Our findings revealed that 10 countries contributed more than 100 publications related to sleep (Table 1). The United States has the highest number of publications, with a staggering 1,804, which is far ahead compared to other countries. China ranks second with 549 publications. The United States has strong research cooperations with China, Japan, Germany, and the United Kingdom (Figure 3A). However, cooperation within Asia, Africa, and South America appears to be weak. The shaped world map (Figure 3B) shows the visual analysis of published regions by VOSviewer software. A total of 3,996 articles related to sleep quality and associated genes research have been published in 92 countries. The world map shows the cooperation relationship strength among countries, and some countries including China, Canada, Australia, Brazil, Japan, and South Korea have limited collaborations with other countries except the United States.

**Table 1:** Countries with more than 100 publications on sleep.

**Rank**	**Country**	**Publications**	**TC**	**TC/ publications**
1	USA	1,804	84,195	46.67
2	China	549	8,876	16.17
3	United Kingdom	402	20,604	51.25
4	Germany	347	12,793	36.87
5	Japan	302	9,828	32.54
6	France	209	8,363	40.01
7	Italy	180	5,812	32.29
8	Canada	175	6,330	36.17
9	Switzerland	150	7,649	50.99
10	Brazil	144	2,857	19.84
11	Netherlands	142	6,181	43.53
12	Spain	130	4,960	38.15
13	Australia	119	3,654	30.71

Regarding institutions, our analysis revealed a substantial amount of collaboration between institutions worldwide. Institutions contributing to sleep research are widely spread out and engage in a broad range of cooperations (Figure 3C). Brigham and Women's Hospital is the most willing to work with other institutions. Pennsylvania University, Harvard University, Stanford University, Yale University, and Minnesota University focus on sleep before 2012 with publications. Broad Institute, Harvard Medical School, Peking University, Zhejiang University, and Manchester University began to study sleep more recently (Figure 3D).

Fourteen institutions contributed more than fifty studies (Table 2). The University of Pennsylvania institution published the highest number of articles, 143, ranking first. This was followed by Minnesota University and Stanford University with 123 and 85 publications, respectively (Table 2).

**Table 2:** Institutions with more than 50 publications on sleep.

**Institution**	**Publications**	**TC**	**TC/ publications**
Pennsylvania University	143	6,641	46.44
Minnesota University	123	6,078	49.41
Stanford University	85	4,575	53.82
California San Francisco University	72	3,050	42.36
Harvard University	71	3,867	54.46
Wisconsin University	70	5,262	75.17
Pittsburgh University	65	2,447	37.65
Johns Hopkins University	63	3,394	53.87
Chicago University	62	3,269	52.73
Fed Sáo Paulo University	58	1,144	19.72
California San Diego University	57	2,345	41.14
Northwestern University	55	5,031	91.47
Surrey University	55	3,833	69.69
Yale University	55	4,732	86.04

### 3.3. Analysis of authors and co-cited authors

**Figure 4:**
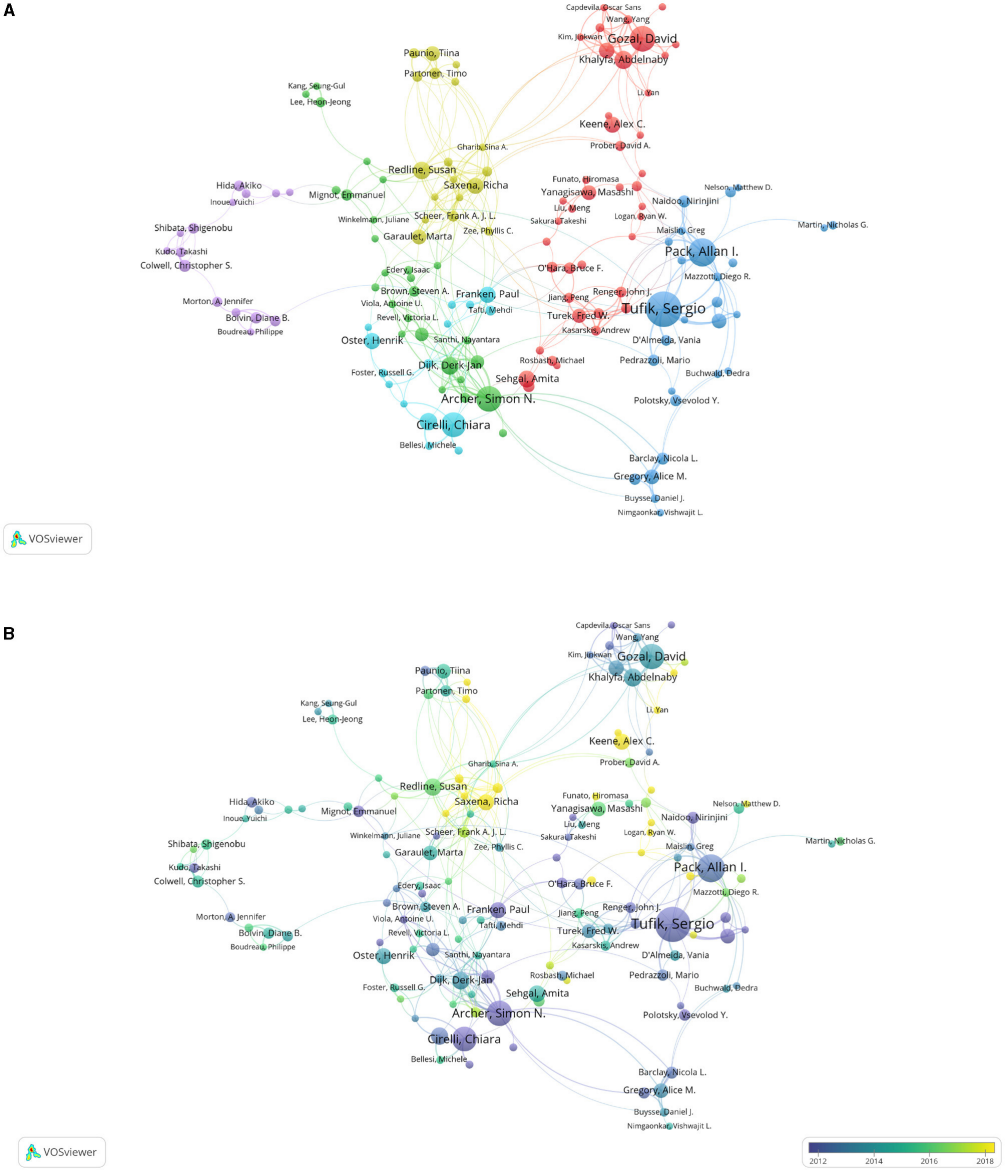
Cooperation maps among authors publishing articles on sleep. **(A)** Cooperation relationships among authors. The size of points means the number of publications. Colors represent clusters automatically calculated by VOSviewer. **(B)** Time trend of cooperation among authors. The colors correspond to the annual time periods of publications.

Partnership among authors promotes global cooperation. A total of 20,615 authors contributed 3,996 articles. Tufik Sergio, Pack Allan I., Archer Simon N., Gozal David, and Cirelli Chiara have published at least 15 articles in this field (Table 3). They study on sleep earlier all before 2014. However, Tufik Sergio has a low ratio of total citations (TC) to publications, while Archer Simon N. and Cirelli Chiara have a high number of citation references with a ratio of more than 60 (Table 3). Saxena Richa, Keene Alex C., Li Yan, and Logan Ryan W. start to focus on sleep in recent years (Figure 4B).

**Table 3:** Authors with more than 15 publications on sleep.

**Authors**	**Publications**	**TC**	**TC/ publications**
Tufik, Sergio	47	920	19.57
Pack, Allan I.	35	1,611	46.03
Archer, Simon N.	31	3,022	97.48
Gozal, David	31	1,243	40.10
Cirelli, Chiara	30	3,360	112.00
Khalyfa, Abdelnaby	20	800	40.00
Dijk, Derk-Jan	19	1,910	100.53
Redline, Susan	19	475	25.00
Tononi, Giulio	19	2,066	108.74
Sehgal, Amita	18	958	53.22
Franken, Paul	17	1,330	78.24
Keene, Alex C.	17	515	30.29
Oster, Henrik	17	845	49.71
Saxena, Richa	17	562	33.06
Kheirandish-Gozal, Leila	16	670	41.88

Most of the studies were conducted by more than one researcher. There seemed to be close cooperation among different authors worldwide. Gozal David and Khalyfa Abdelnaby have the strongest communication. There is no major cluster formed in the map, and the cooperations among authors are various (Figure 4A).

### 3.4. Analysis of journals

**Figure 5:**
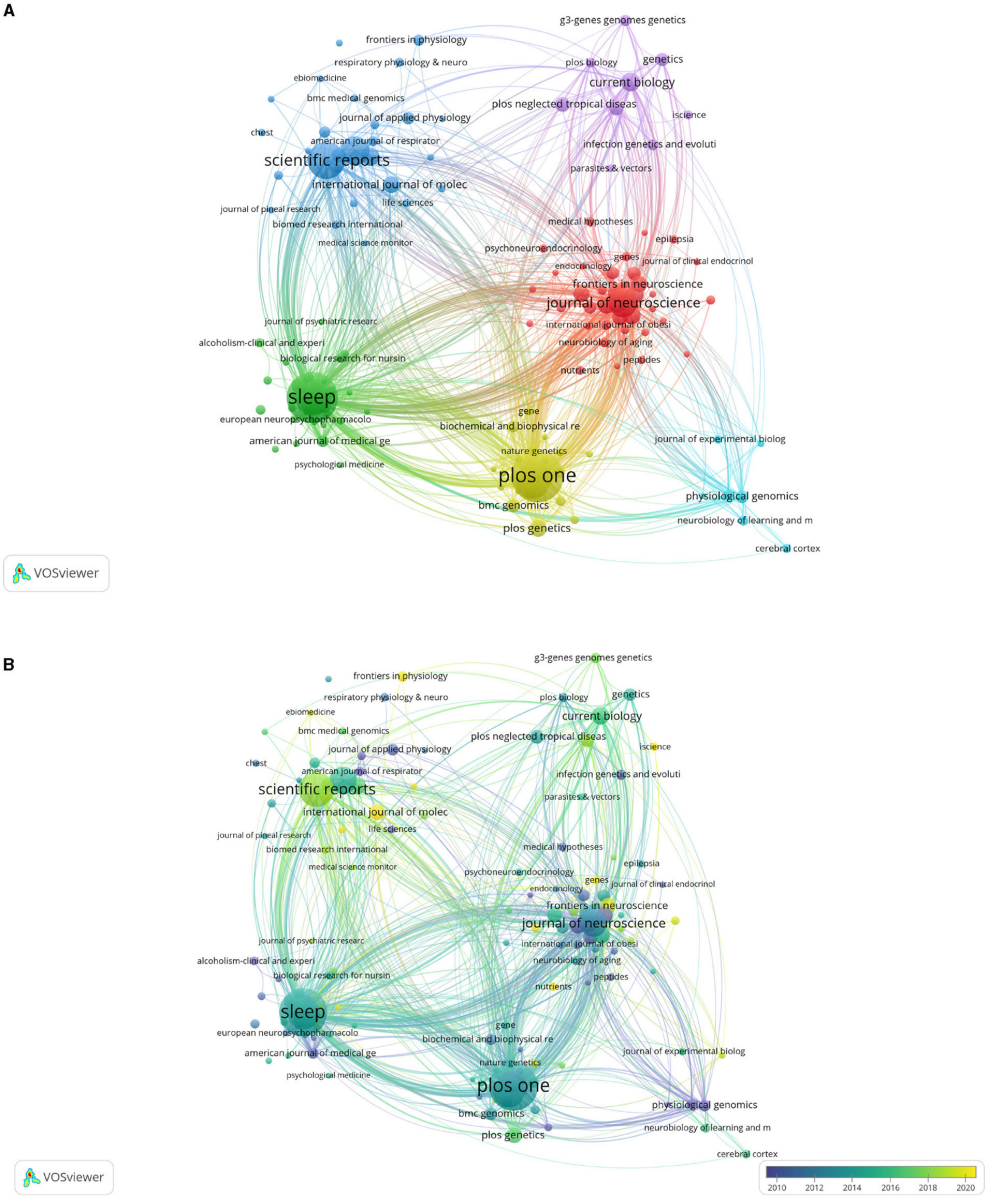
Maps of journals that published articles on sleep. **(A)** Cluster map of journals. The size of points means the number of publications on sleep. Colors represent clusters automatically calculated by VOSviewer. **(B)** Time trend of journals publishing articles on sleep. The colors correspond to the annual time periods of publications.

In total, 147 journals have published 2,497 articles on sleep. The journals with the top three publications are PLoS ONE (164), Sleep (159), and Scientific Reports (96) (Table 4). There are 32 articles related to sleep on Nature, Science, and Cell, but they have the highest ratio of total citations (Table 5). As shown in Figure 5A, the time trend map shows Molecular Therapy, Brain Research, and Neuroscience published articles on sleep earlier with more than 30 publications (Figure 5B). On the contrary, Circadian Clock in Brain Health and Disease, Frontiers in Genetics and iScience start to published research studies on sleep since 2021.

**Table 4:** Journals with more than 30 publications on sleep.

**Journal**	**IF**	**Publications**	**TC**	**TC/publications**
PLoS ONE	3.752	164	999	26.93
Sleep	6.313	159	1,250	41.85
Scientific Reports	4.996	96	482	16.60
Chronobiology International	3.749	84	649	28.46
Proceedings of the National Academy of Sciences of the United States of America	12.779	83	872	103.92
Journal of Neuroscience	6.709	63	476	81.79
Sleep Medicine	4.842	63	245	21.32
Molecular Therapy	12.910	44	810	78.64
Journal of Sleep Research	5.296	36	300	25.42
Behavioral Brain Research	3.352	34	120	22.38
Nucleic Acids Research	19.160	34	443	38.76
Current Biology	10.900	33	368	56.58
Neuroscience	3.708	32	196	35.41
Journal of Biological Rhythms	3.649	31	303	31.03

**Table 5:** Journals with a ratio of TC to publications on sleep >100.

**Journal**	**IF**	**Publications**	**TC**	**TC/publications**
Science	63.714	10	269	454.70
Nature	69.504	15	373	204.00
Cell	66.850	7	173	204.00
PLoS Biology	9.593	12	177	153.83
American Journal of Physiology-Endocrinology and Metabolism	5.900	6	49	151.50
Nature Genetics	41.307	12	283	146.92
Neuron	18.688	20	313	130.80
Proceedings of the National Academy of Sciences of the United States of America	12.779	83	872	103.92
Blood	25.476	7	137	101.57

### 3.5. Analysis of co-cited references

**Figure 6:**
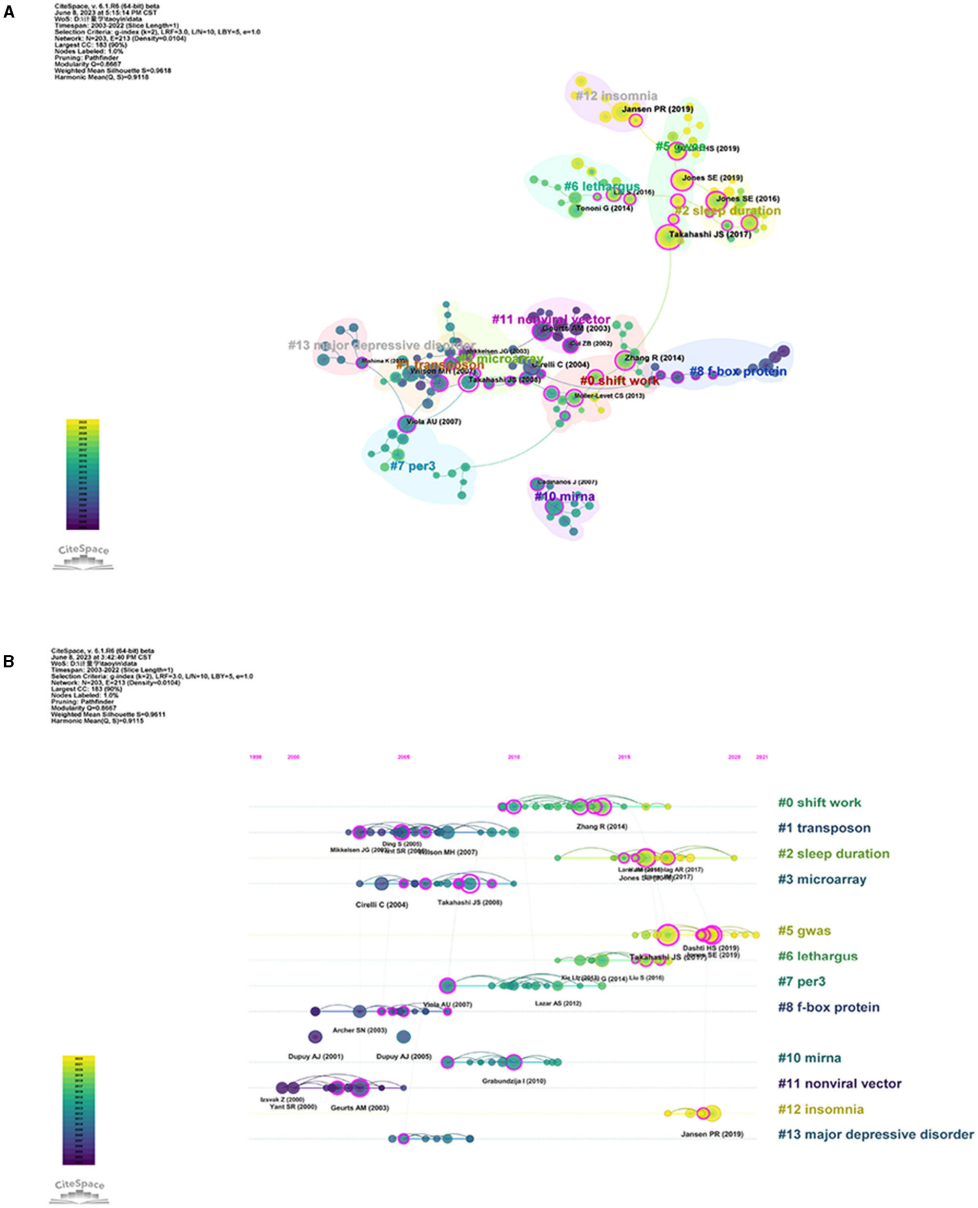
Co-cited references in sleep publications. **(A)** Visualization network of co-cited references regarding sleep articles. The size of points means the number of being cited. The colors padded correspond to the annual time periods of publications. **(B)** Timelines of keywords in co-cited references.

Subsequently, we utilized clustered network analysis to delve deeper into the co-citations and conduct a thorough investigation of their interconnections. As shown in Figure 6A, the co-cited references were clustered into 12 major cluster labels: shift work, transposon, sleep duration, microarray, GWA, lethargus, *per3*, F-box protein, miRNA, non-viral vector, insomnia, and major depressive disorder. A timeline view of the distinct co-citations is shown in Figure 6B to present all the cited literature more clearly. Each ball represents a cited article. The size of the ball is proportional to the number of citations. The connected ball indicates that these articles are cited together. With purple representing relatively old citations and yellow representing more recent citations, we found that in sleep quality and associated genes research, mental illness has been a hot topic since 2005. The study of insomnia and sleep duration is an emerging area of research after 2015 and has received increasing attention in recent years. What is interesting to us is that GWAS has been the focus of recent research, and we look forward to finding more interesting genes related to sleep through this method.

### 3.6. Analysis of keywords

**Figure 7:**
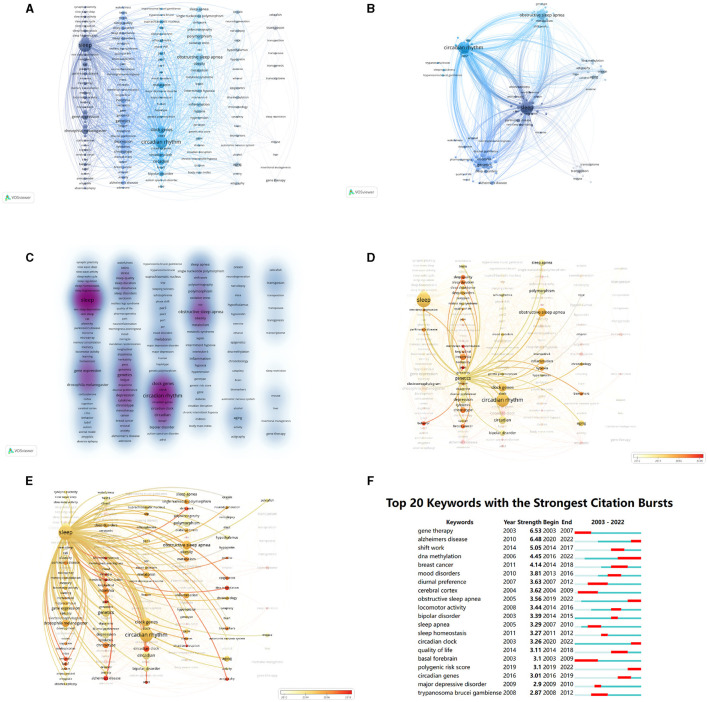
Keywords in sleep publications. **(A)** Visualization network of keywords with more than 10 occurrences. **(B)** Density map of keywords with more than 10 occurrences. **(C)** Cluster map of keywords. The size of points means the occurrences of keywords. The lines mean the relationships between two keywords. Colors represent clusters automatically calculated by VOSviewer. **(D)** Time trend of keywords related to “genetics”. The colors correspond to the annual time periods of publications. **(E)** Time trend of keywords related to “sleep”. The colors correspond to the annual time periods of publications. **(F)** Top 20 keywords with the strongest citation bursts analyzed by CiteSpace. The red areas mean the burst periods of keywords.

Keyword analysis plays a crucial role in identifying research hot spots and trends, offering valuable insights into future directions. The VOSviewer software was used to perform co-occurrence cluster analysis on the keywords of the article. The minimum occurrence times of each keyword were set to 10 and 164 keywords were screened from the 6,973 keywords to form a visual map (Figure 7A). There were six clusters summarized (Figure 7B), including sleep, circadian rhythm, obstructive sleep apnea, genetics, orexin, and transposon.

In the keyword clustering heat map, the color depth is proportional to the number of studies, and the darker the color, the higher the popularity of the keyword, the more studies. “Sleep” and “circadian rhythm” received the most studies (Figure 7C).

By examining the bursts of keywords over time, valuable insights into the research trajectory and emerging areas of focus within the field of study can be gained. Among the identified keywords, “basal forebrain” and “cerebral cortex”, associated with brain regions, emerged as the earliest bursting keywords. Subsequently, keywords such as “diurnal preference,” “shift work,” “mood disorder,” “bipolar disorder,” and “sleep apnea” gained prominence. More recent bursts of interest were observed in keywords such as “Alzheimer's disease,” “DNA methylation,” “circadian clock,” “circadian gene,” “obstructive sleep apnea,” and “polygenic risk score” (Figures 7D, E).

In this study, a comprehensive analysis was conducted on 312 keywords that occurred more than 10 times, yielding noteworthy results. The top five frequently occurring keywords were identified as follows: “circadian rhythm” (185 occurrences), “sleep deprivation” (162 occurrences), “circadian rhythms” (148 occurrences), “obstructive sleep apnea” (106 occurrences), and “clock genes” (89 occurrences). The keywords formed five distinct clusters representing key areas of research interest, including sleep, obstructive sleep apnea, circadian rhythm, genetics, and epilepsy (Figure 7F).

## 4. Discussion

In our study, bibliometric mapping was used to visualize the development of sleep quality and associated genes research from 2002 to 2022. Our findings revealed a continuous increase in scientific output in the field of genetics and sleep over the past two decades. The ranking of publication numbers of countries, universities, authors, and journals helped researchers to search for cooperations, to seek positions, or to publish their works conveniently. Analyzing the collaborative relationships among different countries/regions and institutions can also reflect the academic exchange in this field. Recent advancements in sleep research studies have been facilitated by experimental techniques such as genome-wide association studies (GWAS) analysis. Additionally, newly emergent research hot spots, including obstructive sleep apnea, circadian clock, and circadian genes, were indicated by keyword burst detection. However, despite the substantial amount of research in this area, there was a lack of studies focusing on predicting and clarifying current research hot topics and frontiers. To address this gap, we cataloged the attributes of existing studies and analyzed the results of cluster labeling and burst detection techniques to develop a nuanced understanding of the current state of research and identify promising avenues for future exploration in this field.

GWAS, which is the study of related single nucleotide polymorphisms (SNPs) for a given phenotype with large human populations, have been applied in different sleep subtypes since 2015, especially in sleep duration research studies. SNPs near PAX8 gene, responsible for regulating thyroid-specific genes, were identified in multiple GWAS studies on self-reported and objective sleep duration (9–12). Mutations in this gene were found to potentially increase sleep duration by 2–3 min (13, 14). Vaccinia-related kinase 2 (*VRK2*), associated with schizophrenia (15), was another gene that had been robustly replicated in the previous GWAS (10). GWAS helped scientists identify lots of new intriguing loci related to sleep such as *DRD2, SLC6A3*, or *GABRR1* in the neuron system (11), but more efforts are needed as few novel loci from GWAS are strongly replicated (14).

Apart from SNPs, epigenetic processes including DNA methylation (DNAm), which means the methyl modification of cytosine by DNA methyltransferase, have been implicated in influencing sleep (16). The loss of DNAm of differentially methylated positions showed an association with insufficient sleep in a cross-sectional genome-wide analysis (17). Circadian clock genes were reported to be regulated by DNAm. For example, the expression level of *Per1* was increased during perinatal development by demethylating within the SCN (18), while *Cry2* showed significantly increased methylation in older mice (19). Moreover, researchers have reported that the methylation level of some genes may correlate with sleep disorders. Forkhead Box P3 (*FOXP3*) and endothelial nitric oxide synthase (*eNOS*) gene DNAm levels have been suggested to play a crucial role in pediatric OSA (20). Moreover, DNAm modules in MAPT, which is a key regulator of Tau proteins in the brain, were implicated in sleep duration in children (21). However, there is still an ongoing debate in this area. A meta-analysis in 2022 showed DNA methylation, at birth or in childhood, was not associated with sleep (22). Consequently, there is an urgent need for epigenome-wide association studies to validate the reliability of specific epigenetic alterations in relation to sleep.

Circadian rhythms, which serve as an endogenous time clock, are present in a wide range of living organisms (23). The neuronal network and molecular mechanisms of circadian rhythms were well studied. The core regulatory loop of circadian rhythms was controlled by the transcription factors brain and muscle ARNT-like protein 1 (*BMAL1*) and circadian locomotor output cycles kaput (*CLOCK*), which promoted the expression of period and cryptochrome genes (*Per1, Per2, Cry1*, and *Cry2*). Consequently, the accumulation of heterodimer PER/CRY inhibited their own transcription, forming a self-regulatory feedback loop. This network regulated the expression of countless other genes and was vital for cell physiology and metabolism (24, 25). Recently, some research studies showed these circadian clock genes had multiple functions outside the suprachiasmatic nucleus (SCN), a major core brain region that controls timekeeping, to influence sleep (26). For example, sleep deprivation for 6 h increased the expression of per1 in forebrain lysates (26) and the expression of *per2* in the whole brain, liver, and kidney but not in SCN (27). Moreover, the expression of *Bmal1* in the skeletal muscle was essential and capable of effectively regulating the total amount of sleep (28). As for *Cry1* and *Cry2*, double knockout mice showed increased non-rapid eye movement (non-REM) sleep (29). The molecular mechanism of circadian genes in regulating sleep is still under investigation.

Obstructive sleep apnea (OSA), which is caused by recurrent episodes of upper airway collapse and obstruction during sleep, is a prevalent sleep-disordered breathing (30). It often manifests through symptoms such as loud snoring, nocturnal awakenings, and excessive daytime sleepiness. The prevalence of moderate OSA in the general population ranged from 9 to 38%, with a higher occurrence among men (30). There are many risk factors that affect the OSA, including unmodifiable risk factors like sex, age, and race, and modifiable risk factors such as obesity, endocrine disorders, or obstruction (31). The genetic etiology of OSA was little known. Previous studies using different methods, such as genetic expression analysis and Mendelian randomization analysis, found some genes may be associated with OSA such as *PCNA, PSMC6* (32), *LEPR, MMP-9*, and *GABBR1* (33). Recently, one GWAS study of OSA using FinnGen found high genetic correlations between OSA and obesity (34). Another twin study showed OSA also had a common genetic background with hypertriglyceridemia (35). Moreover, genetic analyses also showed patients with OSA have higher C-reactive protein and tumor necrosis factor-alpha levels (36) and were susceptible to diseases such as atrial fibrillation (37), but the mechanism of these associations was not clear.

Sleep disorders were associated with a higher risk of developing depressed and suicidal thoughts in those with major depressive disorder (MDD). MDD could be influenced by genetic factors, with a heritability of 40–50% based on twin studies. New research studies indicate that depression and sleep disorders share pathogenesis. According to a study conducted by Ma et al., circadian rhythm disturbances might contribute to depression (38). Park et al. utilized a non-parametric model-free0000000000 multivariate dimensionality reduction (MDR) approach and found that circadian genes, *TIMELESS* rs4630333 and *CSNK1E* rs135745, were significantly associated with MDD and bipolar disorder (39). Moreover, genetic variations in adenosine metabolism were associated with depression, sleep, and attention disturbances. *MyD88*, essential for microglial activation, affected microglial homeostasis functions and lowered the serotonergic neuronal output, leading to insomnia and depressive behavior.

Sleep-related disorders are complex phenotypes influenced by both genetic and environmental factors, leading to significant individual variations (40). In contrast, common genetic-related diseases such as albinism and hemophilia are less affected by environmental factors and are easier to study. Genes involved in the regulation of sleep-related disorders are complex, and genetic research in this area is still in its early stages. Many questions remain unanswered. Many other diseases are often associated with sleep-related disorders. Studying the genes related to sleep-related disorders greatly aids in understanding the mechanisms of other diseases, such as mental illnesses and binge eating disorder (BED) (41), which are closely linked to sleep disorders. Unlike other diseases, sleep-related disorders do not affect specific organs, making them more challenging to study (3).

Although many genes related to sleep disorders have been identified through existing technological means, it is difficult to replicate the genes obtained in different laboratories, and many genes cannot be validated. Furthermore, because sleep-related disorders also exhibit environmental susceptibility, it is important to take into account regional and ethnic differences when conducting genetic association analyses (40).

In conclusion, bibliometric analysis offered an objective and quantitative method for assessing publication performance between countries, researchers, and research institutions. Our results showed considerable interest in the field of sleep quality and associated genes research in recent years, particularly the study of GWAS, circadian rhythms, OSA, and depression. The relationship between genes and sleep disorders has gained increasing attention. Recent literature has gradually revealed the specific mechanisms involved, providing potential avenues for prevention, treatment, and intervention of sleep disorders.

## Data availability statement

The original contributions presented in the study are included in the article/supplementary material, further inquiries can be directed to the corresponding author.

## Author contributions

YT: Conceptualization, Data curation, Formal analysis, Investigation, Methodology, Project administration, Software, Visualization, Writing—original draft. YQ: Data curation, Software, Writing—original draft. SC: Conceptualization, Software, Validation, Writing—original draft. TX: Data curation, Writing—review and editing. JL: Formal analysis, Writing—review and editing. DS: Supervision, Writing—review and editing. WY: Supervision, Writing—review and editing. XC: Funding acquisition, Writing—review and editing.
